# Comparative analysis of induced sputum and bronchoalveolar lavage fluid (BALF) profile in asbestos exposed workers

**DOI:** 10.1186/1745-6673-6-23

**Published:** 2011-08-14

**Authors:** Evangelos C Alexopoulos, Demosthenes Bouros, Maria Dimadi, Aneta Serbescu, Giorgos Bakoyannis, Fivos P Kokkinis

**Affiliations:** 1 Occupational Health Unit, Department of Public Health, Medical School, University of Patras, GR-26500 Rio Patras, Greece; 2 Medical School, Athens University, Athens, Greece; 3 Department of Pulmonology, Medical School, Democritus University of Thrace, Greece; 4 Department of Pulmonology, 'SOTIRIA' Chest Hospital, Athens, Greece; 5 Institute of Pulmonology 'M. Nasta', Bucharest, Romania; 6 Pulmonology Clinic, General Hospital of Lamia, Greece

## Abstract

**Background:**

Biological monitoring of healthy workers exposed to hazardous dusts lack validated screening tools. Induced sputum (IS) cellular profile was compared with bronchoalveolar lavage fluid (BALF) profile in asbestos exposed workers in order to assess its usefulness in monitoring workers exposed to asbestos for a long period of time.

**Methods:**

IS and BALF analysis was performed in 39 workers of a car brakes and clutches factory that uses chrysotile asbestos. Selection criteria were an employment history of > 15 years and the absence of a diagnosis of pneumonoconiosis. The type of cells, the existence of dust cells, of iron laden macrophages and of asbestos bodies were assessed and compared between IS and BALF samples.

**Results:**

35 IS samples (90%) had dust cells, 34 (87%) iron laden macrophages and in 8 samples (21%) asbestos bodies were found. In most samples neutrophils were dominated. Samples with asbestos bodies (ABs) had significantly higher lymphocytes and lower neutrophils count compared with the samples without ABs. Macrophages and neutrophils in IS and BALF exhibited significant inter-relations (Spearman's rho: 0.26-0.29, p < 0.05) while IS lymphocytes count showed an inverse relation with BALF neutrophils (Spearman's rho: -0.36). Neutrophils and dust cells were highly correlated between the samples (Spearman's rho: 0.35, p < 0.05) while IS dust cells and lymphocytes were inversely related (Spearman's rho: -0.36, p < 0.05). More years of employment in the company was related with more neutrophils (Spearman's rho: 0.26) and less lymphocytes (Spearman's rho: -0.33) count. In multivariate analysis the presence of AB in IS samples was strongly related to the presence of asbestos bodies and lymphocytes count in BALF samples.

**Conclusions:**

IS and BALF analysis showed a similar cellular profile indicating that IS sampling in exposed workers to asbestos as a less invasive and expensive method may be useful in providing an insight both for inhalation of dusts and inflammatory processes in the lung.

## Background

The occurrence of disease due to occupational exposure to asbestos is well-recognized but surveillance and biological monitoring of exposed workers lack easily implemented tools and techniques. It is based mainly in the traditional tools: occupational past history, x-ray, lung function tests and environmental measurements. The assessment of bronchoalveolar lavage fluid (BALF) has been suggested as a potentially important diagnostic tool in the evaluation of past and present asbestos exposure [1]. The asbestos bodies count and the cellular type of the BALF used to characterize the intensity of asbestos exposure [2-5]. Although it is recognized that further studies are needed to standardize measurement methods and interpretation of values obtained from various biological samples such as sputum, BALF, lung tissue [6,7]. The examination of sputum is a noninvasive method to study particulate burden and inflammatory processes in the lung. Researchers have studied the relevance of asbestos bodies in spontaneous sputum production [8,9]. In a study, a comparison of BAL and IS specimens yielded similar quantitative and qualitative results [10].

In the present study, induced sputum (IS) cellular profile was compared with bronchoalveolar lavage fluid (BALF) profile in workers exposed to asbestos for a long period of time in order to assess in what extent the induced sputum sample analysis provide an insight as far as it concerns the inhalation of dusts and inflammatory processes in the lung.

## Methods

### Study Population

The study population consisted of workers (mainly blue collars) in a Romanian factory building brakes and clutches for cars. Chrysotile asbestos had been used in this factory from its foundation up to December 1999. By the end of 2000, study participants were interviewed at entry into the study. Based to data provided by the occupational health physician, workers were selected if they have completed at least 15 years to worksites with medium to high asbestos exposure intensity (atmospheric levels > 5 fibres per mL) and if they were not diagnosed with pneumoconiosis. Chest × ray films, were interpreted according to the ILO classification of radiographs of pneumoconiosis by two experienced physicians [11]. Subjects with a profusion grade of ≤ 1/0 were considered as not having pneumoconiosis. The occupational physician estimated the possible severity of exposure to asbestos according to occupational history, the specific job title, and the written risk assessment. It was roughly estimated that median cumulative exposure may exceed 150 ((fibres/mL)* yrs) but no further data on exposure measurements have been made available. Thirty nine workers (25 male, 14 female) out of a total of 200 fulfilled the above criteria. All 39 employees were asked to participate in this study by giving their informed consent and agreed to participate. The study was approved by Bucharest Hygiene Institute. All workers have been born and lived in the area near the factory at least as long as they worked in the plant.

Individual data were selected by a questionnaire including questions on age, birth place, resident place, smoking history, duration of employment in current and previous jobs, and on respiratory and other complaints. Intensity of exposure was estimated based on the specific job title, current duties and on worksite risk assessment. Based on that workers were categorized by occupational health department as highly or medium exposed. Questions on complaints of chronic cough, chronic sputum secretion, wheezing, dyspnea, and attacks of chest tightness were also included. The questionnaire also categorized workers to non-smokers (never smoked), current smokers (currently smoking cigarettes, cigars or pipes) and ex smokers (formerly smoked regularly but stopped smoking for at least 1 year before the study). The occupational health investigation was completed by a spirometric lung function test, which was performed with a pneumotachograph spirometer. Measurements and procedures were carried out according to the standards of the European Respiratory and the American Thoracic Societies by qualified occupational nurses [12]. Workers were transported from the factory to the Bucharest University Hospital "Marius Nasta". During a 3-days period, they underwent routine hematological and biochemical tests, chest x-ray, spirometry, and ECG.

### Bronchoscopy and Sputum induction

Fiberoptic bronchoscopy was performed under local anesthesia and was followed by bronchoalveolar lavage (BAL) after obtaining informed consent to bronchoscopy. The bronchial tree was evaluated for endobronchial lesions macroscopically. BAL was performed by infusion of 200 ml saline water (37°C) into the right middle lobe divided in 3 consecutive doses. The lavage was centrifuged at 500 G (1300 r/min) for 10 minutes and it was checked macroscopically following homogenization and filtration so as to remove mucus and then the cellular population was evaluated by cytometry. The total number and the vitality of cells, the existence of dust cells (macrophages with particles), of iron laden macrophages and of asbestos bodies (ferruginous bodies on asbestos cores) with May-Grunwald-Giemsa stain was also assessed. Finally, the specimens were screened for mycobacterium tuberculosis (Ziehl-Nielsen stain) and for existence of cancer cells.

Sputum induction was carried out in the last day of exam. Subjects inhaled nebulized 3.5% saline solution for 10-20 min through a mouthpiece and were asked to cough and expectorate sputum into a sterile plastic container. Following homogenization and filtration so as to remove debris and mucus, the cellular population was evaluated by cytometry [13].

### Statistical Analysis

The principal outcome of the study was sputum cellular type. For categorical variables the chi-square or the Fisher's exact test were used. For comparisons the Kruskal-Wallis and the Mann-Whitney rank sum test were applied. The level of significance was set at 95% (p = 0.05). We also have compared the continuous (BALF) and the categorical (ordinal) variable (IS), by calculating sensitivities, specificities and likelihood ratios for the performance of IS in predicting pathologically high BALF values. The significant corresponding ROC curves and the areas under the ROC curves are also given. Univariate analyses were performed to examine the relation of the covariates age, gender, smoking habit, duration of total employment in the current job with cellular type. Likelihood ratio tests were applied to select the initial variables for inclusion in the multivariate analyses, with, as an inclusion criterion, a level of significance of 0.10. A multivariate model included all variables that contributed significantly to the final model (Wald statistics, criterion of p < 0.05). All statistical analyses were performed with SPSS software (version 17.1.0.).

## Results

### Study population descriptives

The study population consisted of 24 males and 15 females, aged 37 - 53 years, 87% employed for more than 20 years in the company. The demographics, history of smoking, and respiratory functional findings in the study population are shown in Table 1. The data are presented by smoking status due to its significance in the current study.

**Table 1:** Individual characteristics and working experience of the studied population

	Non smokers n = 19		Ex-smokers n = 6		Current smokers n = 14	
Males (n, %)	7	36,8	6	100	11	78,6

Age, years; mean+SD	46,6	4	46	4,5	45,6	4,6

Years of employment; mean ± SD	24,1	4,2	22,8	3,3	23,4	4,5

Respiratory PPE use (n, %)	3	15,8	0		3	21,4

Spirometry						

FVC; mean+SD	86,4	7,1	84	3,4	80,4	11,3

< 80% of pred. FVC (n, %)	1	5,3	0		4	28,6

FEV1; mean+SD	87,8	10,3	87,9	4,9	82	11,5

< 80% of pred. FEV1 (n, %)	1	5,3	0		4	28,6

FEV1/FVC, < 70% (n, %)	1	5,3	0		0	

Asbestos bodies						

in BALF	9	47,4	2	33,3	3	21,4

in IS	6	31,6	0		2	14,3

PPE: personal protective equipment

Smokers had smoked on average 19.14 pack years (sd 9.48) while ex-smokers had stopped at least 2 years before the study and had smoked on average 19.21 pack years (sd 18.7). Four individuals presented obstructive type syndrome, 2 of them were males and 3 smokers. In addition, a non smoker female had a Tiffeneau index of 68%. Most individuals were not aware of asbestos exposure consequences and only six used consistently any respiratory protection before 1999. It is worth mentioning that among those reported to use respiratory protection there was none AB traced either in IS or in BALF.

All smokers and ex-smokers reported cough compared to 58% of non smokers (p = 0.014). Dry cough reported by 21 subjects; 64% among smokers, 50% among ex smokers, and 47% among non smokers. Values of FEV1 and FVC were reduced in smokers while more smokers had no asbestos bodies compared to non smokers but these differences did not reach a statistical significant level (p > 0.05). Workers' IS samples with ABs had higher FEV1 (91.1% vs. 84.4%, p = 0.10) and FVC (89.3% vs. 82.5%, p = 0.05).

### Sputum cellular profile

Cell counts are listed in Table 2. Thirty five samples (90%) had dust cells and thirty four (87%) iron laden macrophages indicating high exposure to dusty environment. Asbestos bodies were found in eight samples (20.5%), 7 out of the 14 workers who had AB in BALF. In most samples neutrophils were dominated.

**Table 2:** Cellular profile of induced sputum samples (n = 39)

	Neutrophils		Lymphocytes		Macrophages*		DC		IL	
**Count (%)**	**N**	**%**	**N**	**%**	**N**	**%**	**n**	**%**	**n**	**%**

< = 5	3	7,7	24	61.6			5	12,9	7	17.9
			
6 - 10	10	25,6	6	15,4	13*	33,4	16	41	16	41

11 - 25	3	7,7	9	23,1	6*	15,4	7	7,9	7	17,9

26 - 40	18	46,2			14*	35,9	10	25,6	7	17,9

> 40	5	12,8			6*	15,4	1	2,6	2	5,1

DC: Dust cells, IL: Iron laden macrophages

*limits in Macrophages is 1-10; 11-22; 23-35 and > 35

Females' samples exhibited higher percentage of macrophages and lymphocytes and fewer dust cells (Table 3). Samples with asbestos bodies had significantly higher lymphocytes count and lower neutrophils count (Table 3). No other significant relation was found between cellular profile of IS and the parameters under study.

**Table 3:** Cellular profile of IS across various characteristics of the studied population

	Neutrophils > 25%		Lymphocytes > 10%		Macrophages > 35%		DC > 25%		IL > 25%	
Gender (n, %)										

Men	15	63	2	8**	0**		11	46**	8	33

Women	8	53	7	47	6	40	0		1	7

Smoker (n, %)										

Current	9	64	2	14	2	14	6	43	5	36

Ex-	4	67	1	17	0		1	17	1	17

No	10	53	6	32	4	21	4	21	3	16

Respiratory PPE (n, %)										

No	21	64	8	24	4	12	10	30	8	24

Yes	2	33	1	17	2	33	1	17	1	17

AB in IS (n, %)										

No	21	68**	5	16*	6	19	8	26	8	26

Yes	2	25	4	50	0		3	38	1	13

Values are n %; Fisher's Exact Test: *p < 0.1; **p < 0.05

Abbreviations: DC: Dust cells, IL: Iron laden macrophages, PPE: personal protective equipment, AB: Asbestos Bodies

### Interrelations of induced sputum and BALF profile

In Table 4 significant correlations between IS and BALF cellular profile are shown. Macrophages and neutrophils among BALF and sputum exhibited inter-relations of borderline significance (Spearman's rho: 0.26 - 0.29) while IS lymphocytes count showed a strong inverse relation with BALF neutrophils (Spearman's rho: -0.36). Neutrophils and dust cells were highly correlated between the samples (Spearman's rho: 0.35). Dust cells in IS were positively related to BALF eosinophil and mast cells (Table 4)

**Table 4:** Correlations (Spearman's rho) among induced sputum (IS) and BALF cellular profile (p < 0.06)

	Induced sputum			
	
BALF	Lymphocytes	Neutrophils	Dust cells	Iron laden macrophages
Neutrophils	-0,36	0,29	0,35	

Eosinophils			0,35	0,43

Mast cells	-0,46		0,29	

Dust cells		0,35		

More years of employment in the company seem to be related with more neutrophils (Spearman's rho: 0.26) but especially with less lymphocytes (Spearman's rho: -0.33). Significant correlations within IS profile included; IS dust cells were inversely related with IS lymphocytes (Spearman's rho: -0.36) and positively related to IS iron laden macrophages (Spearman's rho: 0.48).

**Figure 1:**
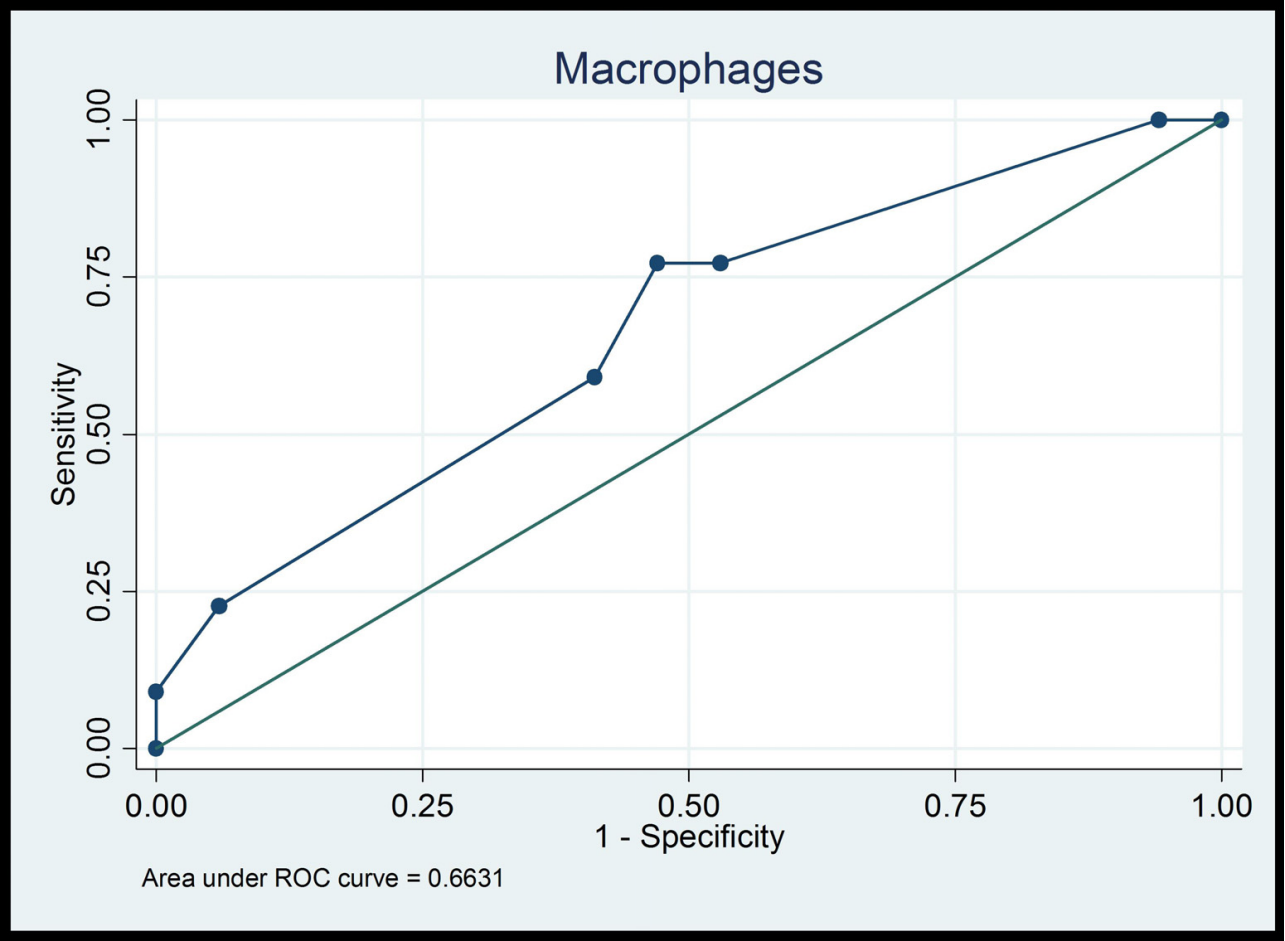
**ROC curve of macrophages in IS and BALF**.

Macrophages in IS predicted satisfactorily high macrophages count in BALF [area (95% CI) under the ROC curve: 0.663 (0.497, 0.829)] (Figure 1). 23% or more macrophages in IS exhibited a sensitivity and a specificity of 77.27% and 52.94% respectively. The corresponding positive and negative likelihood ratios were 1.642 and 0.429 respectively.

**Figure 2:**
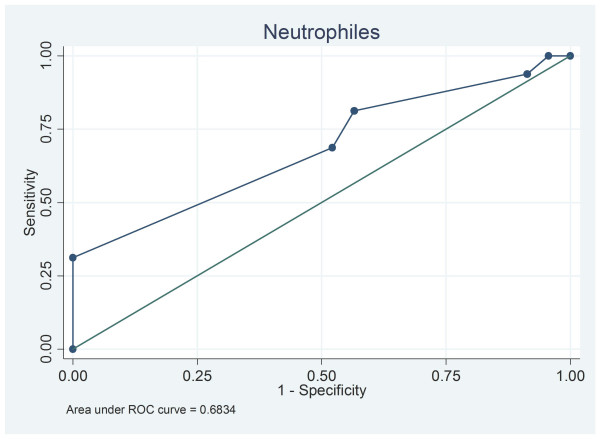
**ROC curve of neutrophils in IS and BALF**.

Neutrophils in IS can predict satisfactorily high neutrophils in BALF [area (95% CI) under the ROC curve: 0.683 (0.515, 0.852)] (Figure 2). 26% or more neutriphiles in IS exhibited a sensitivity and a specificity of 81.25% and 43.48% respectively. The corresponding positive and negative likelihood ratios were 1.438 and 0.431 respectively.

In multivariate analysis (Table 5) we found that the presence of ABs in IS samples was very strongly related to the presence of asbestos bodies in BALF and with more lymphocytes both in BALF and IS while were negatively related with neutrophils in IS. It is worth mentioning that iron laden macrophages in IS exhibited a positive relation with eosinophil count (OR 0.74; 95%CI 0.21 to 1.27) and mast cells count (OR 0.11; 95%CI 0.01 to 0.21) in BALF (linear regression). As far as it concerns IS neutrophiles and lymphocytes count additional relations in multivariate analysis included besides the presence of asbestos bodies, gender and years of employment. A consistent but not significant pattern was evident between use of respiratory protection and the other variables studied (Table 5).

**Table 5:** Multivariate regression analysis on various cellular elements in IS and BALF^

	*INDUCED SPUTUM (IS)*		*BALF*	
	***Neutrophiles (> 10%)*** ***OR (95%CI)***	***Lymphocytes (> 10%)*** ***OR (95%CI)***	***ABs*** ***OR (95%CI)***	***Lymphocytes (> 10%)*** ***OR (95%CI)***

*Females vs. males*	*1,28 (0,27 to 6,16)*	***18,56 (1,60 to 214,89)***	*0,18 (0,02 to 1,66)*	*0,31 (0,07 to 1,39)*

*Employment (in years)*	***1,31 (1,03 to 1,66)***	***0,71 (0,48 to 1,05)***	*0,98 (0,81 to 1,20)*	*1,03 (0,88 to 1,22)*

*Use of respiratory PPE*	*0,21 (0,02 to 1,72)*	*0,43 (0,03 to 5,71)*	0*	*0,75 (0,12 to 4,90)*

*Presence of ABs in IS*	***0,07 (0,01 to 0,63)***	***16,78 (1,08 to 261,63)***	***23,97 (2,51 to 229,02)***	***6,43 (0,96 to 42,87)***

*R Square* *(Nagelkerke)*	*0,38*	*0,50*	*0,36*	*0,37*

^Adjusted for the final model (p < 0,06; in bold), other consistent correlations are also presented; *ABs not found in any reported PPE use

## Discussion

In this study, the cellular profile in samples obtained by two methods (induced sputum and BAL) in workers exposed to chrysotile asbestos was compared. The study demonstrated that samples recovered by induced sputum in workers exposed to asbestos show a similar cellular profile to samples recovered by BAL. Few similar comparative studies have been published for healthy subjects, patients with asthma, chronic bronchitis, and suspected pneumoconiosis [7,10,14,15].

In multivariate analysis, ABs in IS samples was strongly related to the presence of asbestos bodies and lymphocytes count in BALF, although as other reports have shown, far less ABs were identified in IS compared to specimens recovered by BAL [8]. Analysis of BALF in the same setting has suggested that long-lasting exposure to chrysotile asbestos triggers an inflammatory response of the tracheobronchial tree independently of smoking; its type was primarily lymphocytic when asbestos bodies are present otherwise the alveolitis was polymorphonuclear [16].

We also found that IS samples contained a higher percentage of neutrophils and a lower percentage of macrophages compared with the samples recovered by BAL whereas the percentage of lymphocytes exhibited higher relation. These results agree with previous studies and furthermore indicate that IS derived mainly from upper lung (neutrophil-rich secretions), whereas the BAL derived from the macrophages-rich distal alveolar space [7,15,17,18]. The similarity in the pattern of cellular profile between IS and BALF samples indicating the involvement of the same inflammatory process as was also previously shown [19].

In our setting both asbestos exposure and fine dust exposure was encountered, confirmed by the existence of high levels of iron laden macrophages and dust cells in BALF and sputum samples. The interrelations of these factors may have hampered the real influence on specific cellular profiles. In these workers who were exposed for long periods, the presence of iron laden macrophages and dust cells is a marker of both mucociliary clearance and the main defensive phagocytic cell (alveolar macrophages) [20,21].

It is worth mentioning that brake lining workers are one of the few groups formed ferruginous bodies mainly on chrysotile cores opposed to that most ferruginous bodies are formed on one of the amphibole types of asbestos as Dumortier et al. have shown [22].

Limitations of the study include its cross-sectional design which does not permit causal inference, and the size of the study population which is relatively small. It has to be mentioned however, that it is particularly difficult to apply even minimally invasive techniques, such as BAL, without the presence of any indication of disease. Detailed data on exposure were not available but the long employment history and the relatively high estimated exposure possibly provides a homogeneous sample. In our setting, any attempt was not made to study control subjects since the interest was on the comparison of surveillance methods in exposed employees.

## Conclusions

In conclusion, the presence of asbestos bodies and iron laden macrophages in induced sputum is strongly related to BALF cellular type in workers exposed to chrysotile asbestos. It seems that IS analysis reflects the inflammatory response in the bronchoalveolar part of the lung suggesting that the technique may be may be useful in providing an insight both for inhalation of dusts and inflammatory processes in the lung. However its usefulness for screening of workers should be further evaluated because the inflammatory response in our study lacks specificity since it might have been induced asbestos, dust and smoking. Further research is needed to evaluate the hypothesis that the quantitative and qualitative analysis of particles recovered by IS as shown in this study can serve as a method in the periodic health examinations of healthy workers exposed to hazardous dusts.

## Competing interests

The authors declare that they have no competing interests.

## Authors' contributions

ECA contributed to statistics, drafting and revised the manuscript. AS contributed to laboratory analysis and the collection of the data. MD participated in study design and coordination. DB contributed to the writing. GB contributed to statistics. FPK conceived of the study and participated in its design, data collection and coordination. All authors read and approved the final manuscript.
